# Design of a Narrow Band Filter Based on a Photonic Crystal Cavity for CO_2_ Sensing Application

**DOI:** 10.3390/s23104958

**Published:** 2023-05-22

**Authors:** Reyhaneh Jannesari, Thomas Grille, Gerald Stocker, Bernhard Jakoby

**Affiliations:** 1 Institute for Microelectronics and Microsensors, Johannes Kepler University, 4040 Linz, Austria; bernhard.jakoby@jku.at; 2 Infineon Technologies Austria AG, 9520 Villach, Austria; thomas.grille@infineon.com (T.G.); gerald.stocker@infineon.com (G.S.)

**Keywords:** photonic crystal, filter, FDTD, PWE, mid-IR sensor, band gap engineering

## Abstract

This paper investigates the use of a miniaturized filter based on a triangular lattice of holes in a photonic crystal (PhC) slab. The plane wave expansion method (PWE) and finite-difference time-domain (FDTD) techniques were utilized to analyze the dispersion and transmission spectrum, as well as the quality factor and free spectral range (FSR) of the filter. A 3D simulation has demonstrated that for the designed filter, an FSR of more than 550 nm and a quality factor of 873 can be attained by adiabatically coupling light from a slab waveguide into a PhC waveguide. This work designs a filter structure that is implemented into the waveguide and is suitable for a fully integrated sensor. The small size of the device provides a strong potential for the realization of large arrays of independent filters on a single chip. The fully integrated character of this filter has further advantages such as reducing power loss in coupling light from sources to filters and also from filters to waveguides. The ease of fabrication is another benefit of completely integrating the filter.

## 1. Introduction

In recent years, photonic crystals have been extensively studied as a potential technology for lab-on-chip optics due to the proffered benefits, such as the ability to confine light within a small volume, the possibility of integration with waveguides, and the potential to create ultra-high-quality factors in resonators. Photonic crystal applications are extensive, ranging from antennas [1] and polarizers to filters and lasers [2,3,4]. Specifically, the combination of ultra-high-quality factors and ultra-small mode volumes in photonic crystal cavities makes them ideal for use as a narrow bandwidth filter [5,6,7]. This work presents a design for a photonic crystal cavity with a filter application that is incorporated into a fully integrated gas sensor for the mid-infrared (MIR) region. Due to its small size, many of these components can be fabricated on the same chip, thus providing multi-functionality capabilities.

**Figure 1:**
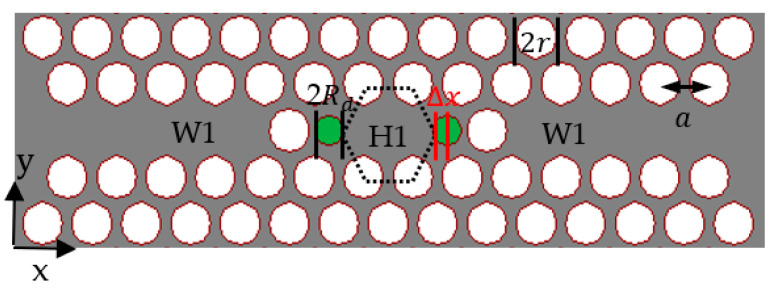
A schematic of the structure. The dotted hexagonal shape represents the center position of air holes in an unmodified H1 cavity. Δx indicates the displacement of two air holes, each with a radius of $R_{d}$. The two slab waveguides mentioned in the text are extended from the tapered regions to the left and to the right, respectively.

Significant efforts have been made to create a filter that has high spectral selectivity, wide spectral tunability, high transmission efficiency, and a small device size [8]. A circular ring positioned adjacent to a rib waveguide has demonstrated great promise as an add-drop-filter (ADF), wherein the forward-propagating wave in the nearby bus waveguide excites a circulating mode in the ring [9]. It is necessary for a silicon ring radius to be smaller than 5 μm in order to achieve a free-spectral range (FSR) of 30 nm in the optical communication window [10]. Nevertheless, the radiation loss of strip-based SOI rings increases rapidly when the size of the ring radius is reduced, which limits the ring radius to a few micrometers in practical applications. Alternatively, photonic crystal structures can address this issue. These structures are based on photonic band gaps rather than total internal reflection in rib waveguides, and thus sharp corners and small radius rings will not lead to higher radiation losses [11]. It has been observed that photonic crystal cavities a few micrometers in size feature excellent characteristics, including low loss, good confinement, and high quality factor, making them excellent filters [12,13]. Photonic crystal ring resonators can be utilized to create optical switches, optical sensors, optical demultiplexers, and ring laser cavities [4,14,15,16,17]. Dideban et al. [18] developed a channel drop filter in a photonic crystal composed of a hexagonal array of circular air rings. The designed filter has a total cavity size of 16 × 14 µm^2^, a Q-factor of more than 5600, and an FSR in the 550 nm region. Although this is good research, the structure is relatively large for the central wavelength of 1.53 µm, and it is also difficult to manufacture. In this research, we concentrated on an add-drop filter utilizing a photonic crystal cavity (H1) to develop a compact filter suitable for integrated photonics. Removing one element from a PhC with a hexagonal periodic array can lead to the formation of an H1 cavity [19]. Coupled mode theory (CMT) can be used to describe the principle of an add-drop filter based on photonic crystals. In this structure, a resonator is placed between two waveguides, one of which acts as a signal bus and the other as a drop. When a pulse enters the bus waveguide, the fields of the guided mode overlap with the resonator. As a result, the resonator and the waveguide become evanescently linked. It is assumed that input and output are arranged symmetrically. If the coupling only has a little impact on the amplitude of the coupled mode, then CMT is an appropriate method for studying the propagation of the light. In [20], a thorough explanation of CMT is provided. The present paper proposes a filter composed of two-line defects serving as the bus and drop waveguide, and a resonant cavity located between the waveguides. The proposed cavity is formed by removing one circular hole from a triangular lattice circular hole. Figure 1 illustrates the schematic of the designed filter. The tuning of the cavity is achieved by varying the size and position of circular holes around the cavity. The band gap is calculated using plane wave expansion (PWE) and the wavelength response of the filter is obtained through the Finite difference time domain (FDTD) technique [21]. In this work, a filter is introduced that has a Free Spectral Range (FSR) that exceeds 550 nm and a size of less than 6 × 6 µm^2^. 

## 2. Materials and Methods

**Figure 2:**
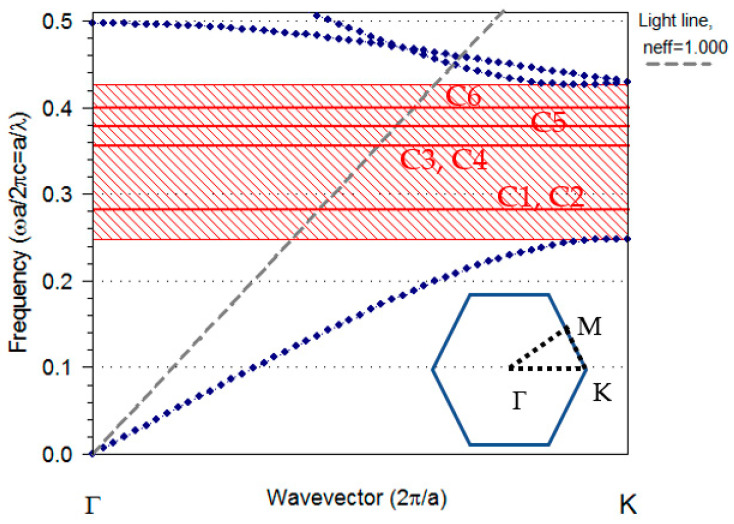
The TE band structure of the H1 cavity modes with r=0.4a (red lines) and dispersion curve of the unaltered PhC (dotted blue lines). The entire shadow area illustrates the photonic band gap of the unaltered crystal. The dashed gray line indicates the light line. Inset, the first Brillouin zone of a triangular lattice is represented by a hexagonal shape. The vertices of this hexagon, which are referred to as high-symmetry points, have Cartesian coordinates set as: Γ(0,0), K$\left(\frac{4\pi }{3a},\;0\right)$, M$\left(\frac{\pi }{a},\;\frac{\pi }{a\sqrt{3}}\right)$.

We consider a PhC made up of a triangular lattice of air holes with a lattice period of a and radius r=0.4a, embedded in a Silicon slab with a refractive index of n= 3.4237. The band diagram of the 2D photonic crystal for the transverse electric TE mode (i.e., modes where the electric field is oscillating in the plane of the PhC slab) is indicated by the dotted blue lines in Figure 2. The shadowed area in Figure 2 indicates a photonic band gap (PBG) in the normalized frequency range a/λ=0.248 to a/λ=0.407 for the photonic crystal structure. The red lines in the PBG present the cavity modes for an unmodified H1 cavity. The modes located above the light line (dashed gray line) are able to “escape” from the photonic crystal, entering the air cladding and forming what is known as leaky modes. The Plane Wave Expansion technique is employed to calculate the propagating modes and photonic band gap of a given structure [21].

**Figure 3:**
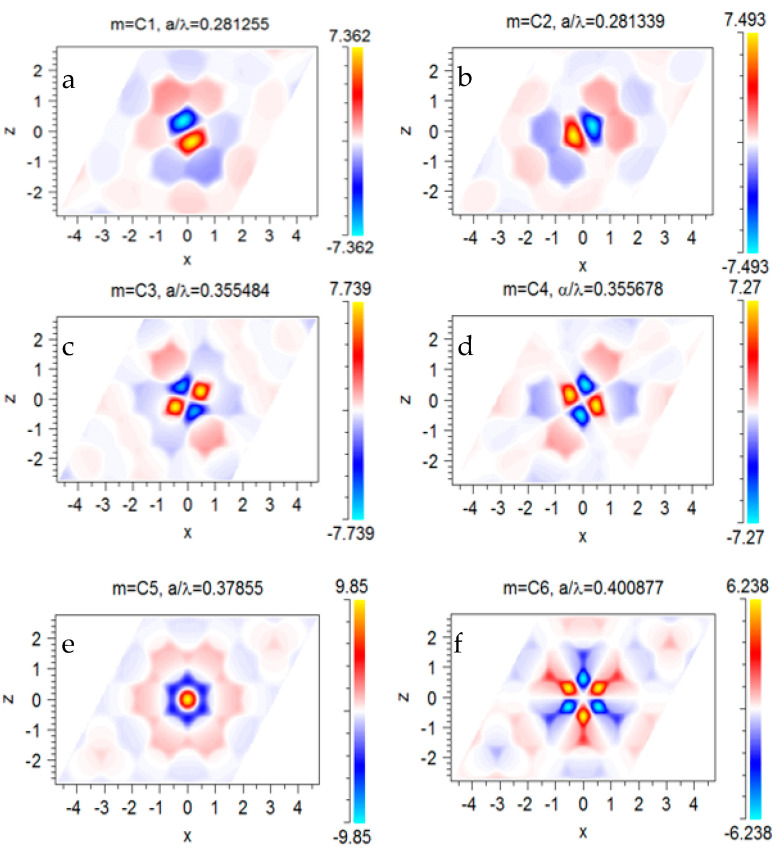
(**a**–**f**) The simulated Hz profile in the x–y plane for the H1 cavity with r=0.4a. Position of the resonant modes in the dispersion diagram marked with C1 to C6 in Figure 2.

The unmodified H1 cavity has six cavity modes—two of which are degenerate with dipole and quadrupole symmetry, respectively, at a/λ=0.281 and a/λ=0.355. Figure 3a–f illustrates the simulated magnetic field profile in terms of the Hz component (perpendicular to the slab surface) of the cavity modes.

The designed filter needs to have a narrow spectral width with a large free spectral range to make it suitable for the gas sensing application. A cavity mode with a symmetry profile distinct from other cavity modes and situated away from photonic band edges can meet these requirements. To reach this condition, the geometrical characteristics of the H1 cavity can be altered to tune the dispersion diagram of the cavity and produce a high-quality factor symmetry mode. This can be accomplished by altering the size and position of the two green holes shown in Figure 1 and finding a mode, which can be coupled to the even photonic crystal waveguide mode. 

This work is part of a research project that entails the development of an infrared absorption spectroscopy-based gas sensor, which is frequently regarded as a particularly effective technology for this purpose. Infrared absorption spectroscopy has a high degree of sensitivity and selectivity due to the unique spectral absorption pattern of the substance being analyzed [22]. The mid-infrared spectrum has been called the “fingerprint” region of molecules because it contains distinctive absorption lines for many gases, including CO_2_, CH_4_, CO, etc. [23,24]. These types of sensors typically have four components: an IR light source, a bandpass filter that selects the target wavelength, an interaction path with the gas, and a detector. We use the detection of CO_2_ through its mid-infrared absorption peak at 4.26 µm as an example for infrared absorption analysis. In addition, the main project utilizes a slab waveguide to detect CO_2_, where the light is transmitted from a broadband source to the slab waveguide [25]. The broadband signal needs to be spectrally filtered to save only the specific fingerprint spectral region (in the case of CO_2_). In this work, we present the design of a filter structure that is implemented into the waveguide and is suitable for a fully integrated sensor. Therefore, the design of this filter is based on the slab mode waveguide, which acts as both an input and an output waveguide. The light propagates through a slab waveguide into a line-defect waveguide (W1), which is created by removing air holes in one row in the ΓΚ direction (inset of Figure 2). From there, the light is directed to the H1 cavity. The parameters of the PhC are selected in such a way that they provide a bandgap of around 4.26 µm. The photonic crystal waveguide (PCWG) supports two modes referred to as even and odd. The even/odd waveguide mode has a symmetric/anti-symmetric pattern in relation to the horizontal centerline of the waveguide. Note that the mismatch in spatial symmetry prevents the antisymmetric modes from coupling to the fundamental slab mode—thus they have no effect on the transmittance [26].

The results of the FDTD method are commonly quite accurate compared to other modelling methods; however, two major problems of the FDTD method are its significant memory requirements and long simulation times. One way to address issues related to memory and time constraints is to utilize the effective index method, although this approach is only applicable to structures with low contrast indices. An alternate strategy is to employ a two-dimensional FDTD model with high-resolution computing to simulate geometry optimization. Even though the absolute values of the outcomes generated by this method may not be valid for the actual structure, the ratios can be utilized in a three-dimensional FDTD model. This approach involves defining the ratios with the help of the 2D model and then applying the 3D FDTD model to obtain the exact geometrical parameters. In the following sections, we will describe and illustrate the model’s key concepts using two-dimensional (2D) simulations. We then present the simulation results of three-dimensional (3D) structures to show that practical devices are achievable.

## 3. Results and Discussion

Using the 2D FDTD method, the effect of varying the geometrical parameters of the cavity on the transmittance spectrum was calculated. A pulse excitation, comprising a Gaussian envelope function multiplied by a sinusoidal carrier, was used to measure the frequency response of the structure. The positions of the monitor were determined inside the cavity to calculate the spectrum of the modes trapped inside the cavity and at the exit of the waveguide to calculate the output field that passed through the filter. For all calculations, the resolution was set to a rectangular grid of a/32 with a=1.276 µm. The boundaries of the simulation domain were enclosed by Perfectly Matched Layers (PML).

**Figure 4:**
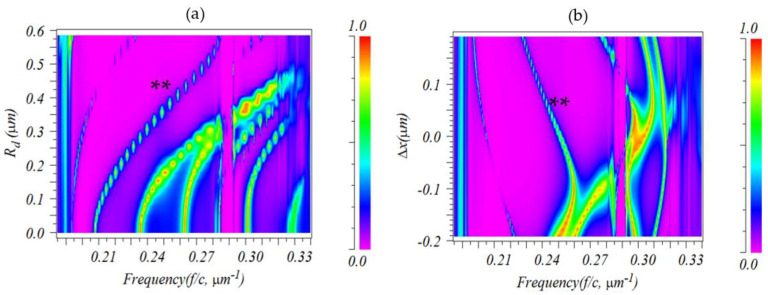
Evolution of the frequency of the H1 cavity modes with alterations of (**a**) the radius $R_{d}$ or (**b**) the displacement Δx of the two holes in the corner of the H1 cavity.

Figure 4 shows the alteration of the cavity modes when the geometry of the H1 cavity was changed. The system was made up of a W1 input, the H1 cavity, and another W1 output. The resonance frequency of the cavity modes in Figure 4a was impacted by the radius of the holes at two corners of the cavity ($R_{d}$; seen in Figure 1). As the radius of the holes increased, the resonance frequencies moved to higher frequencies, but not in a linear proportion. The dispersion diagram presented in Figure 4b displays the impact of the displacement of two holes in the corner of the cavity on the resonant frequency of the cavity modes when $R_{d}=0.37\;µm$. A positive Δx (Δx>0 indicates that the holes were displaced away from the center of the cavity) led to an increased free-standing frequency gap between modes.

**Figure 5:**
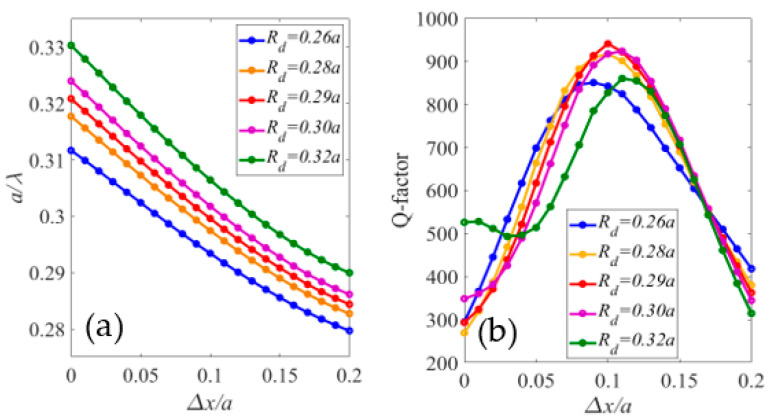
(**a**) Normalized resonate frequency and (**b**) quality factor of the H1 cavity mode marked by “**” in Figure 4.

Analyzing Figure 4 shows the mode indicated by “**” had a large free spectral range with varying $R_{d}$ and Δx; thus, it was chosen for further investigations of its Q-factor. Figure 5a,b shows the dispersion band diagram and quality factor of the mode versus Δx for different $R_{d}\;$values. As Δx increased and $R_{d}$ decreased, the normalized resonance frequency shifted to lower frequencies. At $R_{d}=0.29a$ and Δx=0.1a, the cavity had the highest Q-factor of 945. A two-dimensional finite-difference time-domain method with a perfect match layer (PML) boundary condition was employed for these computations.

**Figure 6:**
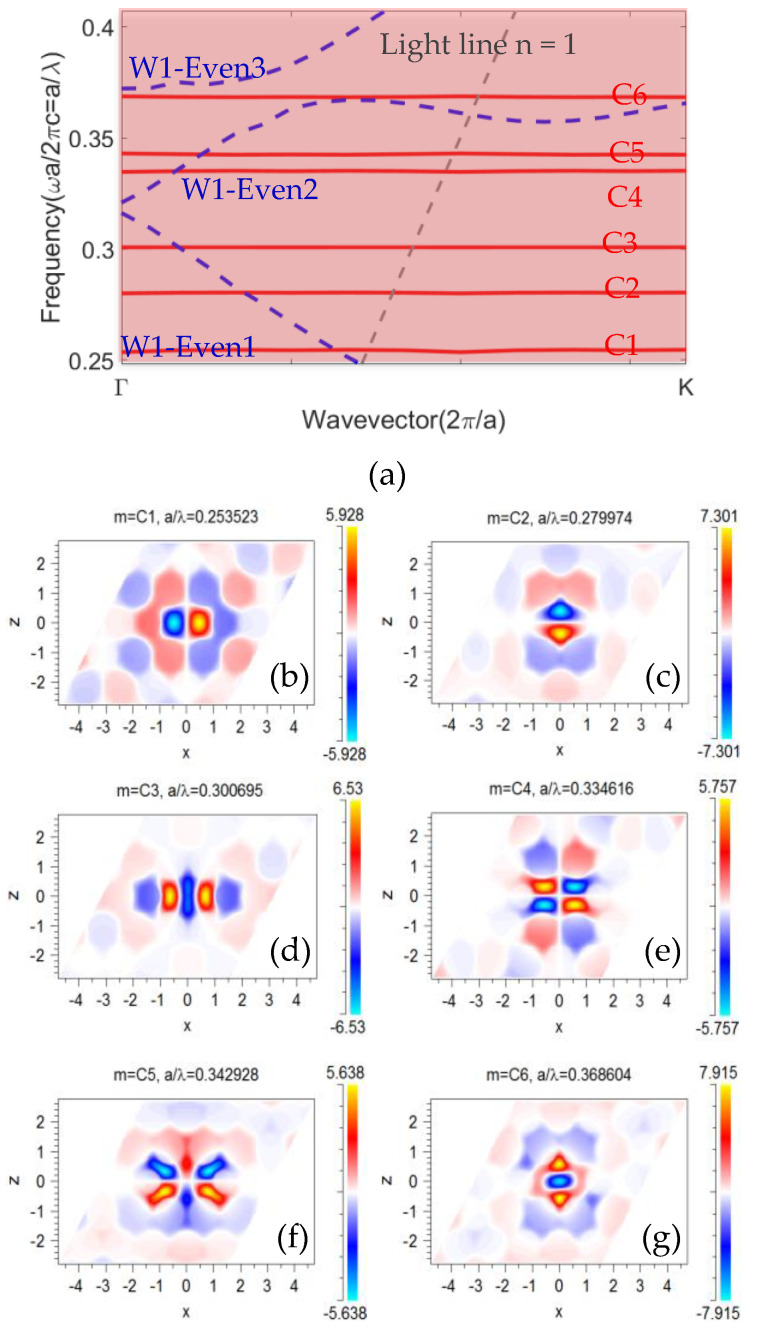
(**a**) The TE band structure of the modified H1 cavity modes with $R_{d}=0.29a$ and Δx=0.1a (red lines) and dispersion curve of the even W1 waveguide (dashed blue line). The entire shadow area illustrates the photonic band gap of the unaltered crystal. The dashed gray line indicates the light line. (**b**–**g**) The simulated Hz profile in the x–y plane for the modified H1 cavity.

The band structure of the modified H1 cavity is presented in Figure 6a. The red lines presented the dispersion band diagram of the modified cavity ($R_{d}=0.29a$, Δx=0.1 a); the even waveguide modes are presented with dashed blue lines. As can be seen, modifying the cavity split the degenerate modes. Figure 6b–g presents the mode profile of the cavity modes in the optimized cavity. The cavity mode with the normalized frequency a/λ=0.3007, labelled C3, was the chosen mode to be optimized as a filter. This mode was very well confined in the cavity area and had even symmetry regarding the horizontal symmetry line (Figure 6d); therefore, it could couple to the waveguide mode. In addition, the dispersion diagram in Figure 6a shows a rather large free spectral range for the C3 mode. Therefore, the C3 mode fulfilled all the criteria necessary for the filter.

**Figure 7:**
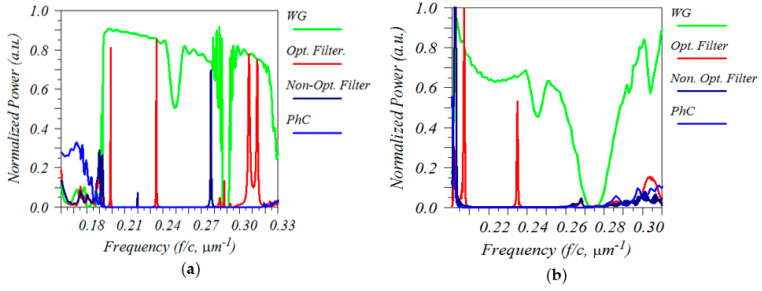
Transmission spectrum of the filter calculated with the (**a**) 2D FDTD or (**b**) 3D FDTD calculation method.

The last step for the 2D calculation was to calculate the transmission spectrum of the structure; this was done by first sending a pulse into the bulk PhC. The blue line in Figure 7a demonstrates the transmittance of the bulk PhC. The transmission was significantly reduced in the frequency range of 0.191–0.315 $\left[{µm}^{-1}\right]$, which was anticipated as the pulse was located within the band gap. The normalized frequency range of 0.243–0.402(a/λ) agreed well with the dispersion diagrams in Figure 2 and Figure 6a. This bandgap was our region of interest because working in this region filtered out most unwanted frequencies. The green line in Figure 7a presents the transmission spectrum of the line-defect waveguide (W1). The W1 waveguide had a large transmission spectrum that encompassed almost the entire band gap. As previously described, the transmission spectrum of W1 was only of the TE mode with even symmetry. The transmission spectra for the optimized and non-optimized filters are represented by red and dark blue lines, respectively. Low transmission efficiency was achieved with a non-optimized cavity (Δx=0 and $R_{d}=0.4a$). However, significant improvements in the transmission efficiency and spectral selectivity at the resonant frequency $1/\lambda =0.234{\;µm}^{-1}$ were observed when the position of the holes in two corners was changed to ±(x+Δx) where Δx=0.1a, and the radius was changed to $R_{d}=0.29a$. Table 1 shows the complete set of geometrical parameters of the simulated structure.

To accurately simulate a photonic crystal structure of holes in a silicon slab, a 3D-FDTD calculation was carried out using a Gaussian pulse source to compute the transmission spectrum and a Perfectly Matched Layer (PML) to set the X, Y, and Z boundaries. For the lateral resolution (in the x, y axes), a rectangular grid of size a/32 was selected, where a was the lattice period. The grid size in the vertical direction (z) was non-uniform, ranging from 100 nm in the air to 10 nm at the interfaces. The pulse was launched into a slab waveguide, coupled to a W1 waveguide, passed through a PhC cavity, and finally sent to an output W1 waveguide and an output slab waveguide. The resolution was set to 25 points per lattice unit a. The slab height and cavity height were both equal to 600 nm. The slab height was chosen to provide the optimum sensitivity for the CO_2_ sensing application. Due to the fully integrated character of this filter, the slab height was taken from the waveguide. To get the maximum Q-factor and FSR, other geometrical parameters of the filter were accordingly tuned. The geometrical parameter for the optimized cavity was set to Δx=0.1a and $R_{d}=0.29a$ and a period of a=1.58 µm. The slab was 0.4a thick and had a refractive index of 3.4237; this allowed for a sharp transmission peak at λ=4.26 µm, which is the absorption peak of CO_2_. However, due to the scalable character of PhCs, by using a suitable lattice constant, the designed cavity could also be scaled to accommodate different wavelengths. The light was strongly confined within the slab by having air claddings on both sides. Figure 7b presents the transmission spectrum of the photonic crystal slab filter. Compared to the 2D structure, the photonic band gap in the 3D structure was smaller and the transmission peak had a lower intensity, which is due to the additional vertical radiational loss of the structure. The 3D FDTD method yielded a Q-factor of over 873 for the designed filter and an FSR of 515 nm and 569 nm for lower and higher wavelengths, respectively. Moreover, due to the strong confinement of the optimized mode within the cavity, the overall lateral size of the filter was reduced to only five lattice periods in the y-direction, resulting in a highly compact structure.

The fabrication of this device was highly CMOS compatible. While this work focused on the design of the device, in the following, we briefly describe an envisaged potential fabrication process. The fabrication covers different processes of deposition, followed by structuring processes in order to pattern the slab. Underneath the slab, a layer of silicon oxide (SiO_2_) is thermally grown in order to decouple the slab from the substrate underneath. The slab itself is poly crystalline silicon, which is deposited as amorphous silicon and recrystallized via a thermal process. For the patterning, a standard photoresist for deep ultraviolet (DUV) lithography is used. After exposure and development, a dry etching process is used to structure the slab. In this respect, DUV lithography is used in order to resolve the needed feature sizes of the PhC.

## 4. Conclusions

In this work, we have demonstrated the design of a narrow-band filter with a wide free spectral range based on a photonic crystal cavity. The photonic crystal is composed of a hexagonal arrangement of air holes in a silicon slab. By precisely adjusting the position and radius of the holes at the corners of the H1 cavity, we were able to optimize the quality factor (Q) and the resonant wavelength of the H1 cavity to achieve high wavelength selectivity. Using 2D and 3D Finite-Difference Time-Domain methods, we examined the normalized transmission spectra of the designed filter. The results of the investigation demonstrated the production of a miniaturized, narrow band filter with a Q-factor of over 870, a size of less than 6 µm, and an FSR of more than 550 nm. The fully integrated filter caused a reduction of radiation loss in coupling light from the broad band source to the filter and further from the filter to the waveguide. Additionally, the small size of the filter makes it an excellent candidate for compact devices or when using a large array of independent filters in a single chip.

## Figures and Tables

**Table 1:** Geometrical parameters of the simulated structure.

	r [nm]	$R_{d}\;\left[nm\right]$	Δx [nm]	a [µm]
2D	510.4	370	127.6	1.276
3D	632	458.2	158	1.58

## Data Availability

Not applicable.
